# Supplementary figures and images for: Transcriptional signatures of clonally derived Toxoplasma tachyzoites reveal novel insights into the expression of a family of surface proteins
Source: PLoS One. 2022 Feb 25;17(2):e0262374. doi: 10.1371/journal.pone.0262374 (PMC8880437; doi:10.1371/journal.pone.0262374)

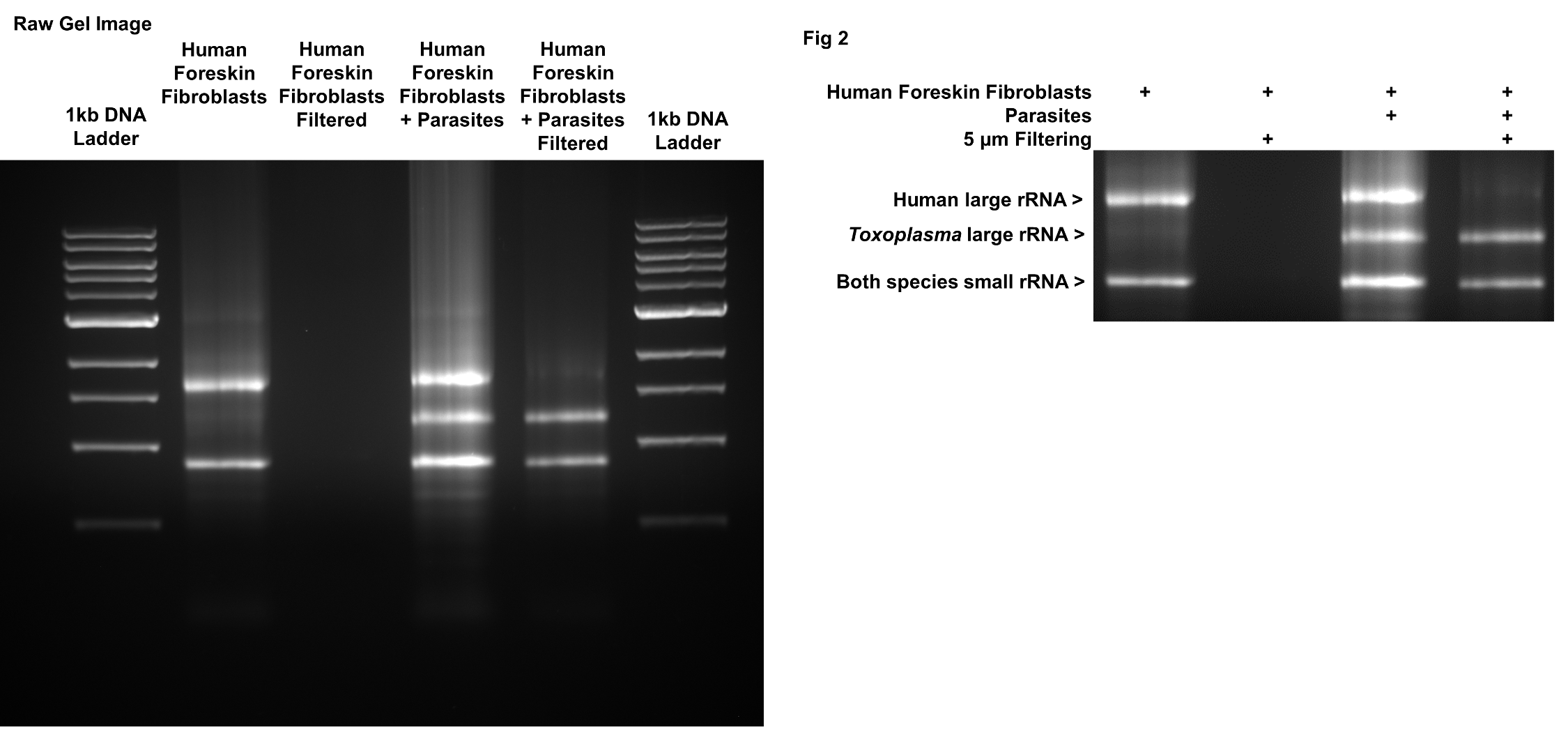

Supplement: S1 Raw images — (TIF) [file pone.0262374.s001.tif]
